# Developmental Exposure to Fluoxetine Modulates the Serotonin System in Hypothalamus

**DOI:** 10.1371/journal.pone.0055053

**Published:** 2013-01-28

**Authors:** Cecilia Berg, Tobias Backström, Svante Winberg, Richard Lindberg, Ingvar Brandt

**Affiliations:** 1 Department of Environmental Toxicology, Uppsala University, Uppsala, Sweden; 2 Department of Neuroscience, Uppsala University, Uppsala, Sweden; 3 Department of Chemistry, Umeå University, Umeå, Sweden; Kent State University, United States of America

## Abstract

The selective serotonin reuptake inhibitor (SSRI) fluoxetine (FLU, Prozac®) is commonly prescribed for depression in pregnant women. This results in SSRI exposure of the developing fetus. However, there are knowledge gaps regarding the impact of SSRI exposure during development. Given the role of serotonin in brain development and its cross-talk with sex hormone function, we investigated effects of developmental exposure to pharmacologically relevant concentrations of FLU (3 and 30 nM (measured)) on brain neurotransmitter levels, gonadal differentiation, aromatase activity in brain and gonads, and the thyroid system, using the *Xenopus tropicalis* model. Tadpoles were chronically exposed (8 weeks) until metamorphosis. At metamorphosis brains were cryosectioned and levels of serotonin, dopamine, norepinephrine, and their metabolites 5-hydroxyindoleacetic acid, 3,4-dihydroxyphenylacetic acid, and homovanillic acid were measured in discrete regions (telencephalon, hypothalamus and the reticular formation) of the cryosections using high-performance liquid chromatography. Exposure to 30 nM FLU increased the concentration of 5-hydroxyindoleacetic acid in hypothalamus compared with controls. FLU exposure did not affect survival, time to metamorphosis, thyroid histology, gonadal sex differentiation, or aromatase activity implying that the effect on the serotonergic neurotransmitter system in the hypothalamus region was specific. The FLU concentration that impacted the serotonin system is lower than the concentration measured in umbilical cord serum, suggesting that the serotonin system of the developing brain is highly sensitive to in utero exposure to FLU. To our knowledge this is the first study showing effects of developmental FLU exposure on brain neurochemistry. Given that SSRIs are present in the aquatic environment the current results warrant further investigation into the neurobehavioral effects of SSRIs in aquatic wildlife.

## Background

Fluoxetine (FLU, Prozac®) is a selective serotonin reuptake inhibitor (SSRI), which increases extracellular serotonin levels and serotonin neurotransmission. It is commonly prescribed for depression, compulsive behaviours, and eating disorders. FLU is one of the SSRIs prescribed to pregnant women. In a study of pregnant women taking FLU the mean serum concentration was 81.4 µg/L and the mean umbilical-cord serum concentration was 60.5 µg/L which implies fetal exposure [1]. FLU is designed to interfere with the serotonin system of the adult brain, and our knowledge on the effects of developmental SSRI exposure is still very limited. Consequently, studies on the effects of FLU on neurotransmitter levels in the brain of developing individuals are highly relevant. Fetal exposure to SSRIs has been associated with impaired intrauterine growth and a number of symptoms indicating altered somatosensory and psychomotor development (reviewed in [2]–[3]). In a recent study, SSRI treatment during pregnancy was associated with pulmonary hypertension of the newborn, a condition that can be life-threatening [4]. It has further been demonstrated that adult rats neonatally exposed to the antidepressants clomipramine and citalopram displayed abnormalities in brain serotonin synthesis, transport and levels [5]–[8]. To the best of our knowledge, effects of developmental FLU exposure on brain neurochemistry are not known.

The serotonin neurotransmission system cross-talks with the sex steroid system and is involved in several aspects of vertebrate reproductive function including sexual behaviour. Estrogen receptors are expressed in serotonergic neurons supporting an interaction between serotonin and estrogen signaling in the central nervous system [9]. Exposure to FLU has been shown to impact the estrogen system by altering plasma estradiol levels and expression of the aromatase gene in the ovary of fish [10]–[12]. The cytochrome P450 enzyme aromatase catalyses conversion of androgens into estrogens and is thought to be involved in sex differentiation of the gonads and brain in lower vertebrates [13] and of the brain in non-human primates and other mammals [14]. Interestingly, it has been shown that developmental exposure of fish to a serotonin synthesis inhibitor (para-chlorophenylalanine) resulted in reduced brain aromatase activity and a female-biased sex ratio, indicating disruption of the gonadal sex differentiation [15]. In a recent study estrogenic effects of FLU were demonstrated also in the rat and in vitro [16]. These data suggest that SSRI exposure, by interference with estrogen synthesis in brain and gonads, could affect reproductive development.

In the present study we investigate impacts of FLU exposure on the developing endocrine system using the African clawed frog, *Xenopus tropicalis*. This species represents an excellent model to study endocrine disruption [17]. First, the organization and components of the amphibian hypothalamus-pituitary-gonadal (HPG) and hypothalamus-pituitary-thyroid (HPT) axes are very similar to those in mammals (reviewed in [18]). Second, the developing estrogen signaling system is very sensitive to chemical disruption, which can be determined by evaluating gonadal differentiation and aromatase activity in brain and gonads in *X. tropicalis* [19]–[20]. Third, the amphibian metamorphosis (transition from larva to frog) is used as a vertebrate model to evaluate chemical impact on the developing thyroid system by assessing endpoints such as thyroid histology and developmental rate [21].

We further explore the *Xenopus* tadpole as a model to investigate effects of FLU on the developing neuroendocrine systems by measuring monoamine concentrations in discrete brain regions. The organization of the brain monoaminergic systems is highly conserved across the vertebrate subphylum [22] even though differences in the organization of the catecholaminergic systems have been reported [23]. However, by applying a segmental approach it is becoming increasingly clear that the organization of central catecholaminergic systems, as well as the general chemoarchitecture and connections of the basal ganglia are largely conserved among tetrapods [23]. In all vertebrates including anuran amphibians, noradrenergic cellbodies are located in the locus coeruleus whereas the most prominent dopaminergic cell groups are found in the substantia nigra and ventral tegmentum of the midbrain, as well as in the diencephalon [23]. Serotonergic neurons are located in the raphe nuclei of the reticular formation [24]–[25]. In addition to this general vertebrate pattern of brain monoaminergic organization, non-mammalian vertebrates also have a group of monoaminergic cells in the hypothalamic periventricular organ, some of which are liquor contacting sending processes into the ventricle [22]–[23], [26].

The specific aim was to determine effects of chronic low exposure to FLU (1.0 and 10 µg/L) on the developing neuroendocrine and endocrine systems during a critical period of development. By using the *X. tropicalis* test system, the experiment served to evaluate effects of developmental SSRI exposure in general, as well as in aquatic organisms exposed to SSRIs in the environment. *X. tropicalis* larvae were exposed to FLU from shortly after hatching until completed metamorphosis, a period that encompasses the early ontogenic development of the HPG and HPT axes, and gonadal sex differentiation [19], [27]. The following endpoints were evaluated: brain monoamine neurotransmitter levels in telencephalon, hypothalamus and the reticular formation, gonadal differentiation, aromatase activity in brain and gonads, thyroid histology and time to metamorphosis. The exposure concentration range was chosen to be relevant both to human exposure and to exposure of aquatic organisms in the environment. We explored the possibility that developmental exposure to pharmacologically relevant FLU concentrations 1) alters neurotransmitter levels in the developing brain, and 2) induces estrogenic effects on the developing reproductive system which may be detected as increased aromatase activity and a perturbed sex differentiation. As FLU previously has been found to cause changes in thyroid stimulating hormone (TSH) levels [28]–[29], we also examined whether FLU exposure could impact the thyroid system in the developing frogs.

## Materials and Methods

### Ethics Statement

All procedures described within were approved by the Uppsala Local Ethics Committee for animal care and use, and were performed in accordance with guiding principles for the care of laboratory animals.

### Animals and Husbandry

*X. tropicalis* tadpoles were obtained by mating three pairs of frogs (Xenopus 1, Dexter, MI, USA). The animals were kept at a 12∶12-h light:dark cycle, in a water temperature of 26.8±0.2°C, and a pH of 7.7±0.2. They were fed Sera micron® (Sera, Heinsberg, Germany). Levels of ammonia and nitrite were measured weekly using standard tests from Merck (Damstadt, Germany).

### Fluoxetine Exposure

Tadpoles were exposed to fluoxetine hydrochloride (FLU, CAS no: 56296-78-7, purity ≥98%) from Nieuwkoop and Faber (NF) stage 47–48 (five days after oviposition) until complete metamorphosis (NF stage 66) [30]. The tadpoles used in the control and exposure groups were drawn from the same population. The nominal concentrations were 0, 3 and 30 nM FLU (corresponding to 1.0 and 10 µg/L). Two replicate tanks were used for each exposure group including the control group. In total, 6 aquaria (8 l) each containing 70 tadpoles were prepared. A semi-static exposure system was used in which 5 liters of the test solution was renewed three times a week during the first two weeks and thereafter daily. FLU was dissolved in ethanol, and the ethanol concentration of was kept at 0.0005% in all aquaria including the controls. Water samples for chemical analysis were collected from the FLU and control aquaria after water change on exposure day 3, 22, and 50.

### Experimental Design and Sampling

As stress can modulate monoamine levels [31], we minimized handling stress by habituating all frogs to handling every day during the exposure period. Aromatase activity was analysed in brain and gonads attached to the kidneys (gonad/kidney complex) at NF stage 56, during the period of gonadal differentiation, and at NF stage 66 when metamorphosis and gonadal differentiation is completed in *X. tropicalis*. At NF stage 56, thirty tadpoles per exposure group (fifteen per replicate) were randomly collected and their brains and gonad/kidney complexes were pooled into 3 pools of 10 brains and 3 pools of 10 gonad/kidney complexes per exposure group.

At stage 66 the frogs were killed and the body length and weight, time to completion of metamorphosis (from oviposition to NF stage 66), and survival rate were recorded. The frogs were dissected using a dissection microscope and sexed by examining gross gonadal morphology. Some frogs could not be unambiguously sexed using gross morphology. Therefore histological sexing of the gonads was performed on 12–17 individuals per replicate. Six to nine individuals per replicate were randomly selected for neurotransmitter analysis. Brain and gonadal-kidney complexes from another five individuals per replicate were sampled for aromatase activity analysis. Five individuals per exposure group were sampled for thyroid histology evaluation.

### Tissue Preservation

The animals were killed in 0.5% benzocaine-ethanol solution (Sigma-Aldrich, St. Louis, MO, USA). For gonadal sex determination, gonad/kidney complexes were excised and fixed in formaldehyde (4% in phosphate buffer). For evaluation of thyroid histology the anterior part of the frogs were fixed in formaldehyde. For neurotransmitter analyses frog heads were frozen in Sakura Tissue-Tek OCT Compound (GENTAUR Belgium BVBA, Kampenhout, Belgium) on dry ice. For the aromatase assay the brain and gonad/kidney complex were frozen in liquid nitrogen and stored in −80°C.

### Histological Processing and Histomorphometry

The fixed tissue was transferred to 70% ethanol. Following dehydration in increasing concentrations of ethanol, the tissue was embedded in hydroxyethyl methacrylate (Leica Historesin, Heidelberg, Germany). Sections (2 µm) from the gonad/kidney complexes were stained with haematoxylin-eosin.

The thyroid glands were sectioned at six levels 50 µm apart and stained with toluidine blue. For each individual (n = 5) the largest cross section was photographed and analysed morphometrically. Thyroidal cross section area and area of the three largest follicles were measured. All follicles per thyroid gland cross section were counted and the epithelial height was measured at 4 points (0, 90, 180 och 270°) of the three largest follicles and a mean value per individual was calculated.

Photographs of the histological sections were taken using a Leica leitz DMRXE microscope equipped with a Hamamatsu ORCA III M digital camera and Openlab 3.09 software (Improvision, Coventry, UK). Histomorphometrical measurements were made using Image J software (Rasband WS, ImageJ, U.S. National Institute of Health, Bethesda, USA).

### Monoamine Analysis and Turnover Ratios

Serial sections (200 µm) from the frozen heads were cut on a cryostat microtome and mounted on glass slides. From 1–2 sections per individual samples were taken in telencephalon, the hypothalamus and the reticular formation using a punch (diameter 0.8 mm, AgnTho’s AB, Lidingö, Sweden). The regions were identified using a neuroanatomical atlas [32]. The whole region was excised by taking 1–3 punches per section. The punched samples were homogenized in 70 µl of ice-cold 4% perchloric acid containing 40 ng/ml dihydroxybenzamine as internal standard. The samples were spun at 13000 rpm for 10 min at 4°C and the supernatants were analyzed for dopamine (DA), norepinephrine (NE), and serotonin (5-hydroxytryptamine, 5-HT) and their metabolites 3,4-dihydroxyphenylacetic acid (DOPAC), 5-hydroxyindoleacetic acid (5-HIAA) and homovanillic acid (HVA) using high performance liquid chromatography with electrochemical detection as described in [33] after being stored at −80°C for less than 5 days. Concentrations were compared to standard solutions and corrected by the internal standard using HPLC software (CSW, DataApex Ltd., the Czech Republic). Protein content was measured in the homogenate pellet using a BCA Protein Assay Kit (Thermo Fisher Scientific Inc., Rockford, IL, USA). The concentration of monoamines and their metabolites was expressed as µg/g protein. The ratios of metabolite to parent monoamine concentration: 5-HIAA to 5-HT, and HVA or DOPAC to DA were calculated as indirect indices of serotonergic and dopaminergic turnover, respectively.

### Aromatase Activity

Aromatase activity was analyzed as described in [34] using a modified method of the tritiated water-release assay [35]. The principle of the assay is the formation of tritiated water via aromatization of the substrate 1β-[^3^H] androstendione. The tissue was homogenized and incubated with 1β-[^3^H] androstendione (spec. act. 23.5 Ci/mmol, PerkinElmer Life and Analytical Sciences, Boston, MA, USA). All samples were prepared in duplicates. The radioactivity of the produced tritiated water was measured for 20 min/sample in a liquid scintillation analyser (Tri-Carb 1900CA, Packard Instrument Co). The determined radioactivity in the tissue samples was corrected for the tritium found in the blanks and a mean value of the duplicates was calculated. Protein concentrations were measured using a BCA Protein Assay Kit (Thermo Fisher Scientific Inc., Rockford, IL, USA). The aromatase activity was expressed as the number of fmol androstendione used per mg protein per hour.

### Determination of Fluoxetine Concentrations in Water Samples

The water samples were filtered through 0.45 µm MF™-membrane filters (Millipore, Sundbyberg, Sweden) prior to acidification to pH 3 (sulphuric acid). Solid phase extraction columns Oasis HLB (Waters Corporation, Milford, MA, USA) were conditioned and equilibrated by 5.0 ml of methanol and 5.0 ml of deionized water. The isotopically labeled internal standard fluoxetine-d_5_ (Sigma–Aldrich, St. Louis, MO, USA) was added (500 ng) to 100 ml portions of each sample and. The samples were passed through the columns at a flow rate of 5 ml min^−1^ and the sample extracts were injected into the liquid chromatography–tandem mass spectrometry (LC–MS/MS) system, described in [36]. The following *m*/*z* transitions were monitored: fluoxetine, 310.3 → 147.8; and fluoxetine-d_5_ was 315.3 → 152.8. Internal standard calibration was used for all samples and the limit of quantification of fluoxetine was 50 ng/L (1.6 nM).

### Statistics

The exposure groups were compared with respect to aromatase activity, thyroid histomorphometry, time to metamorphosis, body weight, and snout-vent length using Kruskal-Wallis test with Dunn’s multiple comparison test (GraphPad Prism 5.0, GraphPad Software, San Diego, CA, USA). Survival rates for the exposure groups were compared using the Chi-Square test (GraphPad Prism 5). Data for the statistical analysis of monoamine levels was log-transformed to fulfill the assumption of normal distribution. To analyse effects of FLU exposure on the monoamine concentration the data were subjected to analysis of variance (ANOVA) followed by the Tukey post hoc analysis (SYSTAT 8.0, Cranes Software International Ltd, Chicago, IL, USA). For all endpoints (except aromatase activity in tadpoles and thyroid histomorphometrical data which represent data from both replicates), data from the two replicate tanks were statistically compared before the data were pooled into one data set per exposure group.

## Results

### Fluoxetine Concentrations

The measured FLU concentrations (mean ±SD) were 0.60±0.01 and 0.70±0.07 µg/L (1.7 and 2.0 nM) in the two 3-nM replicates, and 9.7±2.7 and 9.5±0.8 µg/L (28.0 and 27.5 nM) in the 30-nM replicates. FLU was not detected in the control aquaria.

### Tadpole Development, Metamorphosis, and Water Quality

Tadpole survival, growth and development did not differ between exposure groups. The survival rates were 81, 88, and 88%, in the control, 3 nM FLU, and 30 nM FLU groups, respectively. Time to metamorphosis was (mean ±SD) 52±12 days in the control group, 54±11 days in the 3 nM FLU group, and 51±9 days in the 30 nM FLU group. The body weight and snout-vent length at metamorphosis did not differ between groups (data not shown). Mean concentration of ammonia in the control, 3 nM FLU, and 30 nM FLU groups were 0.07, 0.09, and 0.10 mg/L, respectively. Mean concentration of nitrite in the control, 3 nM FLU, and 30 nM FLU groups were 1.06, 0.12, and 0.15, mg/L, respectively. All in all, there were no indications of any general adverse effects of the testing environment on the health of the tadpoles.

### Sex Ratios

The frequency of females in the 3 nM FLU, 30 nM FLU, and control groups were 19/29, 14/30, and 10/24, respectively, and did not differ significantly.

### Monoamine Levels and Turnover Ratios

Results from monoamine analyses are presented for each tank replicate in Table 1. As no sex difference in neurotransmitter levels were recorded, the data represent mixed-sex groups. Neither were there any significant differences between the tank replicates in the control nor in the FLU exposure groups with respect to neurotransmitter levels. These data were consequently pooled into one set of data per exposure group for the statistical analysis of differences between exposure groups.

**Table 1:** Monoamine concentration and ratios of metabolite to parent monoamine in three different brain regions of *Xenopus tropicalis* frogs at completed metamorphosis, after developmental chronic exposure to 0, 3 or 30 nM fluoxetine (Control, 3 FLU, and 30 FLU) in duplicate tanks (A and B).

	NE (µg/gprotein)	DOPAC (µg/g protein)	5-HIAA (µg/g protein)	HVA (µg/gprotein)	DA (µg/g protein)	5-HT (µg/gprotein)	DOPAC/DA *10^−3^	HVA/DA * 10^−3^	5-HIAA/5-HT * 10^−3^
*Telencephalon*									
Control A	3.51±1.40 (6)	2.19±1.30 (6)	2.54±2.21 (6)	1.94±0.39 (6)	12.62±16.04 (6)	6.06±4.34 (6)	477±614 (6)	329±224 (6)	410±349 (6)
Control B	8.64±5.22 (4)	9.07±10.18 (4)	8.00±7.14 (4)	2.70±3.58 (4)	56.91±67.70 (4)	17.78±18.82 (4)	406±691 (4)	66±52 (4)	695±558 (4)
3 FLU A	3.63±2.22 (6)	1.49±1.26 (6)	3.23±2.35 (6)	2.11±1.23 (6)	16.35±9.21 (6)	15.29±14.57 (6)	139±152 (6)	272±372 (6)	469±465 (6)
3 FLU B	5.94±4.86 (5)	3.61±3.07 (6)	3.15±1.23 (6)	2.60±2.03 (6)	18.35±10.30 (6)	8.10±8.96 (6)	215±141 (6)	242±288 (6)	1760±1669 (6)
30 FLU A	4.38±3.88 (5)	1.19±0.81 (5)	10.31±15.13 (6)	1.92±1.69 (6)	17.98±13.25 (6)	22.54±33.12 (6)	149. ±114 (5)	222±251 (6)	1080±1475 (6)
30 FLU B	6.70±5.85 (8)	8.47±11.93 (8)	15.63±34.05 (8)	3.25±3.01 (8)	31.28±30.43 (8)	14.45±15.10 (8)	407±696 (8)	145±85 (8)	1037±1241 (8)
*Hypothalamus*									
Control A	4.84±4.88 (6)	7.58±9.20 (6)	3.19±3.00 (6)	3.25±2.76 (6)	13.56±16.10 (6)	17.19±23.60 (6)	607±443 (6)	354±207 (6)	474±491 (6)
Control B	3.75±2.06 (5)	2.22±1.35 (5)	4.07±4.72 (5)	1.26±0.64 (5)	14.85±7.45 (5)	6.94±7.45 (5)	1966±169 (5)	96±61 (5)	876±908 (5)
3 FLU A	2.65±2.19 (6)	2.63±2.25 (6)	3.51±0.53 (6)	2.14±0.94 (6)	15.37±15.65 (6)	12.39±11.50 (6)	272±248 (6)	330±395 (6)	542±413 (6)
3 FLU B	5.84±5.00 (6)	7.14±4.52 (6)	4.00±3.11 (6)	1.69±0.65 (6)	22.66±19.25 (6)	9.33±10.32 (6)	570±669 (6)	144±151 (6)	1461±1300 (6)
30 FLU A	4.48±3.00 (8)	10.67±13.47 (8)	7.95±6.29 (8)	1.62±1.07 (8)	18.11±10.94 (8)	22.12±21.93 (8)	592±480 (8)	100±58 (8)	448±270 (8)
30 FLU B	4.56±2.49(7)	4.94±4.11 (8)	12.87±20.78 (8)	10.65±17.83 (8)	42.90±79.34 (8)	22.50±42.92 (8)	321±281 (8)	284±250 (8)	836±476 (8)
*Reticular formation*									
Control A	12.85±26.12 (6)	4.93±3.64 (6)	7.81±13.60 (6)	3.05±1.41 (6)	16.70±27.71 (6)	13.66±18.13 (6)	922±1099 (6)	445±313 (6)	1751±2957 (6)
Control B	4.14±2.37 (6)	9.75±13.44 (6)	9.51±14.11 (6)	3.38±2.73 (6)	29.38±24.32 (6)	15.55±25.38 (6)	429±428 (6)	284±447 (6)	1871±2321 (6)
3 FLU A	8.47±10.42 (6)	8.07±12.91 (6)	7.46±8.30 (6)	3.95±3.26 (6)	48.74±77.08 (6)	24.19±35.39 (6)	529±664 (6)	559±901 (6)	385±202 (6)
3 FLU B	2.85±2.70 (5)	7.19±7.52 (5)	3.94±3.12 (5)	1.43±0.88 (5)	8.46±2.93 (5)	7.15±6.80 (5)	1042±1362 (5)	219±188 (5)	771±832 (5)
30 FLU A	2.11±1.447 (7)	6.54±6.45 (7)	3.30±4.81 (7)	1.24±0.64 (7)	11.49±14.92 (7)	5.18±8.63 (7)	807±972 (7)	316±398 (7)	6574±14693 (7)
30 FLU B	2.34±1.11 (8)	10.69±13.89 (8)	3.62±4.14 (8)	2.61±0.95 (8)	17.04±23.00 (8)	6.28±9.61 (8)	707±721 (8)	325±186 (8)	1760±2566 (8)

Values represent mean ± SD (n).

Abbreviations: Norepinephrine (NE), 3,4-dihydroxyphenylacetic acid (DOPAC), 5-hydroxyindoleacetic acid (5-HIAA), homovanillic acid (HVA), dopamine (DA), and serotonin (5-HT).

**Figure 1:**
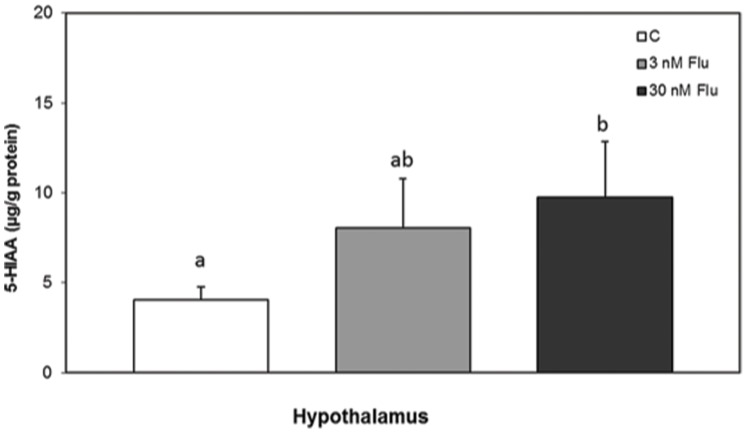
Concentration of 5-HIAA in the hypothalamus of *Xenopus tropicalis* at metamorphosis after developmental chronic exposure to fluoxetine at two different concentrations, 3 nM FLU (grey bar) and 30 nM FLU (black bar) and in controls (C, white bar). Values represent mean ± SEM. Different superscript letters indicate significant differences between exposure groups (P<0.05, Tukey post hoc). n = 12−18.

Exposure to 30 nM FLU significantly elevated the 5-HIAA concentration in the hypothalamus region compared to controls (Fig. 1, ANOVA, F_2, 36_ = 4.099, P = 0.025, Tukey post hoc, P = 0.019). In FLU exposed frogs, the mean levels of both serotonin and 5-HIAA in the reticular formation were lower than in the control group, but due to a large individual variation this difference could not be statistically ascertained (Table 1).

### Aromatase Activity

There were no differences between the exposure groups in brain or gonadal aromatase activity, neither at tadpole stage 56 (data not shown) nor at metamorphosis (Table 2).

**Table 2:** Aromatase activity (mean ± SD) in gonads and brain of *Xenopus tropicalis* at completed metamorphosis after developmental chronic exposure to 0, 3 or 30 nM fluoxetine (Control, 3 nM FLU, and 30 nM FLU).

	Aromatase activity at metamorphosis			
	(fmol/h/mg protein)			
Exposuregroup	Brain		Gonads	
	Females^a^	Males^b^	Females^c^	Males^d^
**Control**	51±31	40±42	8.6±2.4	4.8±1.1
**3 nM FLU**	53±50	54±30	9.2±2.1	4.8±1.9
**30 nM FLU**	38±36	24±20	8.7±2.0	4.5±1.0

a n = 8−10.

b n = 10.

c n = 10 ovary-kidney complexes.

d n = 3–6 testis-kidney complexes.

### Thyroid Gland Histology

Analysis of thyroid gland histology revealed no differences between the exposure groups. The mean (±SD) thyroid gland cross-sectional area and follicular area were 0.08 (±0.001) and 0.013 (±0.004) mm^2^, respectively. The mean (±SD) number of follicles per cross section were 10.3 (±4.1) and follicular epithelium height was 9.8 (±2.4) µm.

## Discussion

The present study shows that chronic exposure to FLU during development impacts the serotonergic system in the developing brain. Considering that FLU is frequently prescribed to pregnant women [37]–[39], the finding that the developing serotonergic system was affected at FLU concentrations lower than the mean measured concentration in umbilical cord serum is of particular interest. FLU exposure had no observed effect on the other neurotransmitter levels, the endpoints for estrogen and thyroid system perturbation or on overall health, indicating a specific effect on the serotonergic neurotransmitter system in the hypothalamus region. Exposure occurred during the beginning of the ontogenic development of the HPG and HPT axes [27]. In mammals including humans these processes occur during fetal development [40]. To the best of our knowledge this is the first study to show that developmental SSRI exposure modulates neurotransmitter levels during the period of early ontogenic development of the HPG/HPT axes. Given the knowledge gaps regarding the impact of SSRI exposure in utero our findings are highly relevant.

The observed increased level of 5-HIAA after developmental FLU exposure is quite opposite to the expected pharmacological effect in adult individuals. In adults, reduced reuptake of serotonin will lead to reduced levels of 5-HIAA as serotonin is metabolised in the pre-synaptic neuron after reuptake. Our finding is consistent with research showing that FLU may have paradoxical effects in the brain of developing animals compared with those in adult individuals [3]. The consequences of fetal exposure to SSRIs are not sufficiently understood, although there is emerging evidence suggesting severe side-effects in children (reviewed in [2]–[3]). In children exposed prenatally to SSRIs, altered stress responses and behavior [41]–[42], blunted somatosensory responses [43], poor psychomotor development [44], increased risk of admission to neonatal intensive care [39], impaired intrauterine growth, increased cord blood levels of thyroid stimulating hormone [29], reduced cord blood level of 5-HIAA, and an array of serotonergic symptoms [45] have been observed. Intrauterine exposure to SSRIs has thus been associated with neonatal changes compatible with serotonergic overstimulation. It should be emphasized that we examined healthy animals that were not subjected to conditions simulating maternal depression or stress. Maternal stress, which has been shown to influence the actions of developmental SSRI exposure using murine models [46], was therefore not accounted for in the present study design.

The reason for the increased concentration of the serotonin metabolite 5-HIAA in the hypothalamus region in the FLU exposed frogs is presently unclear but may indicate an increased serotonin release in this region. Acute effects of SSRI treatment involve inhibition of serotonin synaptic re-uptake, reduced serotonin catabolism, and hence a decrease in 5-HIAA concentrations. However, long-term SSRI treatment is known to desensitize and attenuate the function of the somato-dendritic serotonin receptors (5-HT1A) in the mammalian raphe nucleus. As these receptors exert a negative feedback action on serotonin neurotransmission, their desensitization will result in increased serotonin release [47]. Thus, it is possible that the chronic FLU exposure applied in the current experiment resulted in a reduced function of 5-HT1A autoreceptors in the raphe which in turn resulted in an increase in the release of serotonin in hypothalamic areas. Adult rats neonatally exposed to non-selective monoamine reuptake inhibitors have been shown to have reduced levels of serotonin and 5-HIAA in the hypothalamus [5]–[6]. Long-term consequences of developmental exposure to SSRIs on the monoaminergic system in frogs remain to be elucidated.

The raphe nuclei of the reticular formation and their efferent projections are considered major anatomical targets for SSRIs in the brain. In rats, neonatal exposure for two weeks to the SSRI citalopram results in reductions in both the rate-limiting serotonin synthetic enzyme (tryptophan hydroxylase) in dorsal raphe and in serotonin transporter expression in the cortex that persisted into adulthood [8]. In the present study the mean levels of both serotonin and 5-HIAA in the reticular formation of FLU exposed frogs were lower than in the control group, but due to a large individual variation this difference could not be statistically ascertained. This variation is probably mainly due to disturbance of the animals during sampling. Stress is known to result in a rapid activation of the brain serotonergic system. Thus, even though the animals were habituated to handling, sampling is likely to have induced a stress response.

FLU exposure had no observed effect on the endpoints for estrogen system perturbation: sex differentiation and aromatase activity. This is in contrast to recent research demonstrating estrogenic effects of FLU in the rat uterotrophic assay and in vitro [16]. In fish it has been shown that FLU concentrations in the range of 0.1–30 µg/L affect the estrogen system by altering plasma estradiol levels and expression of the aromatase gene in the ovary [10]–[12]. Neither could we detect any effects on the endpoints for thyroid system toxicity: time to complete metamorphosis and thyroid gland histology. These negative results indicate that in the *Xenopus* tadpole the serotonergic neurotransmitter system in the brain was a specific target for FLU exposure. Altogether, the results of the current study indicate that regional neurotransmitter levels in the brain of *X. tropicalis* may serve as sensitive endpoints for neurodevelopmental disruption.

Many pharmaceuticals pass through the sewage treatment plants and eventually enter the aquatic environment. The lowest FLU concentration in the present study (0.6 µg/L) is comparable to concentrations FLU and other SSRIs detected in water bodies receiving sewage effluent [48]–[50]. The total concentration of SSRI compounds is higher than that for individual compounds and has been measured to 3.2 µg*/*L in close proximity to wastewater effluents [51]. FLU and other antidepressants have been detected in wild fish [52]. Recently, it was shown that an environmentally relevant FLU concentration (0.5 µg/L) reduced milt volume in fish [14], indicating that this type of compounds may pose a risk to wild fish. To the best of our knowledge, information on effects of SSRIs on the developing neuroendocrine system in amphibians is lacking. The present results warrant further investigation into the neurodevelopmental effects of SSRIs in aquatic wildlife.

In conclusion, the present study demonstrates for the first time that developmental exposure to FLU modulates the serotonin system during a critical period of brain development. FLU exposure had no observed effect on the other neurotransmitter levels or on the endpoints for estrogen and thyroid system perturbation, indicating specific changes in the serotonergic neurotransmitter system in the developing brain. Considering that fluoxetine is frequently prescribed to pregnant women and that the consequences of fetal SSRI exposure are not sufficiently understood, the observation that the developing serotonergic system was affected at FLU concentrations found in umbilical cord serum is of particular interest. The present results increase our knowledge on impacts of SSRIs on neurochemistry during a critical period of brain development and they support the contention that the developing serotonergic system is highly sensitive to impacts from exposure to SSRIs.
